# Effect of six weeks of blood flow restriction combined with Tabata training on anaerobic capacity in male badminton players

**DOI:** 10.3389/fphys.2025.1656050

**Published:** 2025-11-07

**Authors:** Yuancheng Xia, Xin Zheng, Kaixiang Zhou, Leyi Jiang, Bojun Song, Shuxian Xu, Zhangyuting Shang, Jin Dai

**Affiliations:** 1 Sports Coaching College, Beijing Sport University, Beijing, China; 2 College of Physical Education and Health Science, Chongqing Normal University, Chongqing, China; 3 School of Strength and Conditioning Training, Beijing Sport University, Beijing, China; 4 College of Physical Education and Health Management, Chongqing University of Education, Chongqing, China

**Keywords:** blood flow restriction, Tabata training, anaerobic capacity, badminton players, lower-limb explosive strength

## Abstract

**Background:**

Blood flow restriction (BFR) combined with high-intensity interval training (HIIT) is increasingly recognized as an effective strategy for enhancing aerobic capacity and muscle strength in athletes. However, there is no consensus on the effects of BFR combined with HIIT on anaerobic capacity.

**Objective:**

This study aims to examine the effects of BFR combined with Tabata training (BFR-Tabata), a type of HIIT, on anaerobic capacity in male badminton players.

**Methods:**

Thirty male badminton players (age: 20.4 ± 1.2 years) were randomized to the BFR-Tabata group (n = 15) or the Tabata group (n = 15). Both groups performed 6 weeks (3 times per week) of Tabata training (20 s maximal effort/10 s rest x 8 sets x 4 rounds). Pre and post-intervention assessments included a 30-s Badminton-specific endurance test, countermovement jump (CMJ), squat jump (SJ), and Wingate anaerobic test (peak power [PP], average power [AP], fatigue index [FI], time to peak [TTP]). A two-way repeated measures ANOVA (time × group) was used to analyze training effects, with Bonferroni *post hoc* tests. Effect sizes were reported as partial eta squared ( $\eta_{\text{p}}^{2}$ ) or Cohen’s d, with significance set at p < 0.05.

**Results:**

The BFR-Tabata training intervention significantly improved anaerobic endurance among male badminton athletes. In comparison to the Tabata group, the BFR-Tabata group exhibited statistically significant differences in badminton-specific endurance (p = 0.02, d = 0.92, moderate effect) and multiple anaerobic performance indicators assessed by the Wingate test, including PP (p = 0.01, d = 1.11, moderate effect), AP (p < 0.01, d = 1.51, large effect), and TTP (p < 0.01, d = 2.10, very large). However, no statistically significant difference was observed in lower limb explosive strength measures, including CMJ (p = 0.50, d = 0.25, small effect), SJ (p = 0.56, d = 0.21, small effect), or during the Wingate test the FI (F = 0.138, p = 0.71, $\eta_{\text{p}}^{2}$ = 0.005, trivial effect).

**Conclusion:**

Blood flow restriction combined with Tabata training is an effective strategy for improving anaerobic capacity in male badminton players, but it has limited enhancement of lower-limb explosive strength.

## 1 Introduction

Badminton is a high-intensity intermittent event characterized by rapid changes of direction, acceleration-deceleration, and vertical jumps over short periods (usually 20 s) during a competition (Edel et al., 2024; Hong et al., 2014; Phomsoupha and Laffaye, 2015; Shariff et al., 2009; Yuan et al., 2025). It also requires a high level of physical fitness, as athletes are given only short breaks between matches (Zhang et al., 2016). The factors that affect badminton performance include not only cardiorespiratory endurance, agility, and quickness, but also anaerobic energy system (Ko et al., 2021). This system supplies energy that helps badminton players sustain a high-intensity effort or maintain their pace, especially when recovery time is limited (Phomsoupha and Laffaye, 2015; Göktepe, 2007). A study reported that in competitive games, badminton players reach over 90% of HRmax, and about 30% of the energy comes from the anaerobic system (Phomsoupha and Laffaye, 2015). High-Intensity Interval Training (HIIT) is recognized as a highly effective training method for enhancing anaerobic performance in players (Franchini et al., 2019). This training method improves anaerobic metabolism by increasing energy expenditure during exercise sessions and elevating excess post-exercise oxygen consumption (EPOC) (BaiQuan et al., 2025). Additionally, HIIT significantly enhances skeletal muscle oxidative capacity and substrate utilization efficiency by promoting metabolic adaptations (Hoshino et al., 2016). Donie et al. have shown that a 6-week HIIT program significantly improved the 30-m sprint in collegiate badminton players (Donie et al., 2021). Ko et al., 2021 demonstrated that 4 weeks of HIIT (three sessions per week) significantly enhanced the anaerobic capacity of badminton players (Ko et al., 2021). Samsir reported a statistically significant improvement in the 20 m shuttle run test following a 10-week HIIT intervention in 16 male adolescent badminton players (Suppiah et al., 2019). Tabata training, a type of HIIT training, is commonly structured as eight repeated cycles of 20 s of maximal-intensity exercise followed by 10 s of rest, resulting in a complete session duration of only 4 minutes. One study has found that Tabata training (20s sprints at 170% V̇O_2max_ with 10 s rest) led to a significant 28% enhancement in maximal accumulated oxygen deficit (MAOD) among male physical students (Tabata et al., 1996). Compared to traditional HIIT, which typically requires 20 min or more to elicit comparable physiological responses, Tabata training induces substantial metabolic and cardiovascular adaptations within a significantly shorter time frame (Tabata, 2019). This time-efficient method is particularly advantageous for badminton players, who require rapid energy turnover, repeated high-intensity efforts, and efficient use of limited training time. A previous review suggested that Tabata training is effective in improving anaerobic endurance in elite athletes; however, it has limited effects on muscle strength and peak power output (Tabata, 2019). To achieve better results without extending the session duration, it is essential to update the current Tabata training methods.

One potential method is to combine blood flow restriction (BFR) with Tabata training. BFR training involves using devices like blood pressure cuffs or elastic wraps on the proximal limb muscles to restrict arterial blood flow during exercise, slowing venous return (Patterson et al., 2019). The principal physiological mechanisms underlying BFR training include the induction of localized muscular hypoxia and the accumulation of metabolic byproducts, notably lactate, due to controlled reductions in muscular perfusion (Freitas et al., 2021). Such physiological alterations activate metabolic and neuromuscular signaling pathways, ultimately enhancing protein synthesis and augmenting metabolic adaptations (Castilla-López et al., 2022). A number of studies have shown that BFR combined with low-intensity aerobic exercises or resistance training effectively improves aerobic capacity and muscle strength in athletes (Chang et al., 2022; Lavigne et al., 2024). These studies reported that such BFR interventions could enhance oxidative enzyme activity, increase V̇O_2max_, prolong time to exhaustion, and promote both muscle hypertrophy and strength (Abe et al., 2010; Scott et al., 2017).

However, the effects of BFR combined with HIIT (BFRIT) on anaerobic capacity are still controversial. For example, Amani-Shalamzari et al. (2020) and Behringer et al. (2017) reported that BFRIT significantly improved V̇O_2max_, anaerobic running capacity, and sprint performance (Amani-Shalamzari et al., 2020; Behringer et al., 2017). Conversely, other studies have suggested that 4–5 weeks of BFRIT fail to improve 30-s Wingate anaerobic power (Held et al., 2020; Mitchell et al., 2019). A systematic review by Chua et al. (2022) indicated that the enhancement of anaerobic capacity by BFRIT was affected by training level, training intensity, and the specific training program (Chua et al., 2022). To our knowledge, no study has investigated the effects of blood flow restriction combined with Tabata training (BFR-Tabata) on anaerobic capacity in male badminton players.

This study aimed to investigate the effects of BFR-Tabata training on the anaerobic capacity of male badminton players, comparing it to traditional Tabata training. The hypotheses were as follows: compared to Tabata, 1) BFR-Tabata training significantly improves anaerobic capacity (peak power [PP], average power [AP], fatigue index [FI], time to peak [TTP]) in male badminton players; 2) BFR-Tabata training significantly improves lower-limb explosive strength (squat jump [SJ] and countermovement jump [CMJ]).

## 2 Materials and methods

### 2.1 Participants

Thirty male collegiate badminton players (age: 20.4 ± 1.2 years; body mass: 72.6 ± 3.2 kg; height: 178.5 ± 7.2 cm) participated in this study. Participants were randomly assigned to either the BFR-Tabata group (n = 15) or the Tabata group (n = 15). All participants were healthy, non-smokers, and not taking any medications or supplements. Eligible male players had competed in the quarterfinals of the National Junior Competition, achieving a top-six ranking in individual events or a top-two ranking in team events at the provincial level or higher. The study was approved by the Ethics Committee of Sports Science Experiment of Beijing Sport University (No. 2025158H), and all procedures were conducted in accordance with the Declaration of Helsinki. Before the experiment, participants were informed of the benefits and potential risks related to the study, and all signed the informed consent form.

Sample size estimation was based on a study by Amani-Shalamzari et al. (2020), which reported an effect size of 0.66 for anaerobic performance (peak power [PP]) (Amani-Shalamzari et al., 2020). Since anaerobic capacity was the primary outcome, power analysis relied on PP. Using G*Power, we determined that 14 participants (7 per group) were needed for a significance level (α = 0.05) and a statistical power of 0.95. To account for potential dropouts and ensure reliable analyses across outcomes such as the vertical jump and badminton-specific endurance tests, we recruited a total of 30 participants.

### 2.2 Study design

**FIGURE 1:**
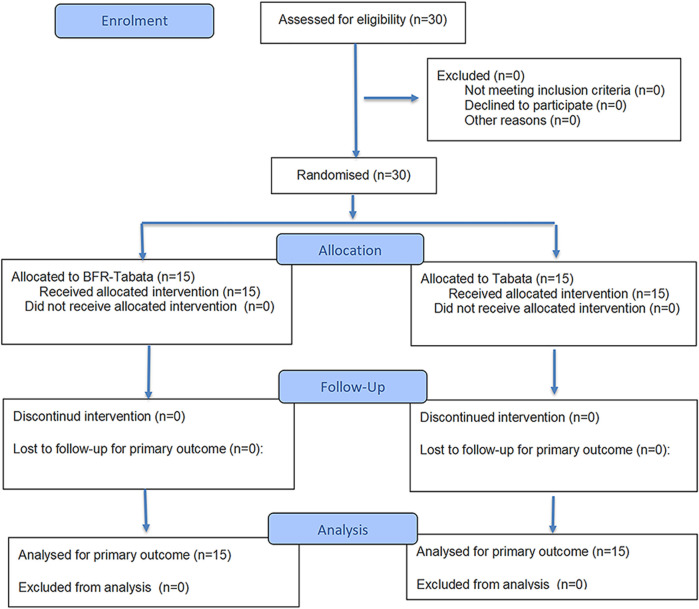
CONSORT flow diagram.

**FIGURE 2:**
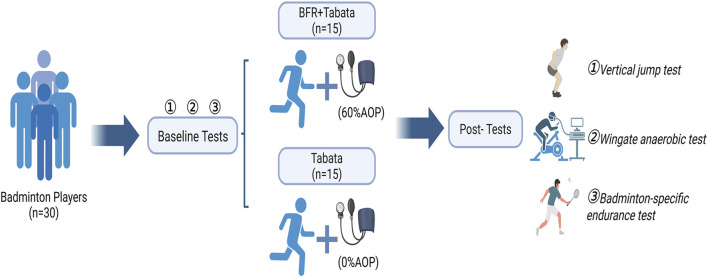
Experimental procedure. BFR, Blood flow restriction; AOP, arterial occlusion pressure.

This study used a 6-week, single-masked (assessor-blind) randomized controlled trial (Figure 1). Participants were randomly assigned to either the BFR-Tabata group or the Tabata group using a computer-generated block randomization sequence (block size = 4). The allocation sequence was generated by an investigator not involved in participant recruitment or assessment, and the assignments were concealed until interventions were implemented. Outcome assessors were blinded to group allocation. The BFR-Tabata and Tabata groups completed the training protocol thrice weekly, allowing for a 24–48-h recovery period between sessions. Training sessions were scheduled on Mondays, Wednesdays, and Fridays from 12:10 to 12:40 p.m. or 3:30 to 4:00 p.m. All participants were required to complete a familiarization session to learn the Tabata training protocol and the testing procedures before the intervention. A certified strength and conditioning coach delivered standardized instruction to ensure consistency in the training and evaluation process. To ensure measurement consistency and minimize potential bias, all evaluations were conducted by the same trained assessor throughout the study. All evaluation sessions were conducted at the same time of day for each participant to prevent potential confounding effects related to circadian rhythms. Temperature (21.2 °C ± 0.3 °C) and humidity (29.0% ± 0.4%) were maintained consistently. Participants were instructed to avoid vigorous exercise for 48 h before each evaluation day and refrain from consuming alcohol and caffeine for 24 h before the sessions (Figure 2). All experimental equipment, including the Doppler ultrasonography (Apogee 1,000), bicycle ergometer (Model 894E, Monark, Sweden), and force platform (Kistler Group, Switzerland), was calibrated before each testing session. Calibration procedures were strictly followed according to the manufacturer’s guidelines to ensure measurement accuracy and reliability.

#### 2.2.1 Training protocol

**FIGURE 3:**
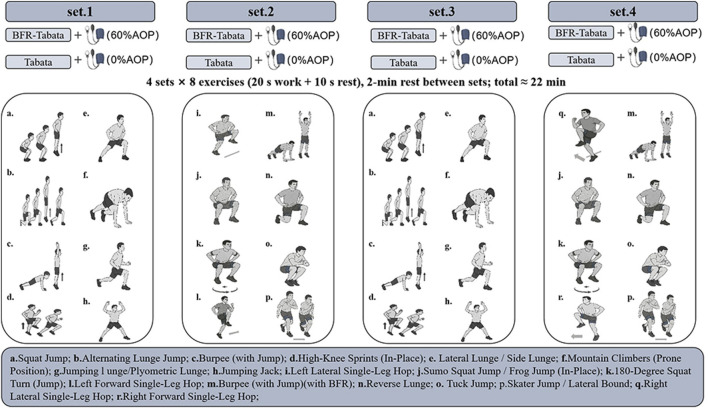
Training protocol. BFR, Blood flow restriction; AOP, arterial occlusion pressure.

Each Tabata session consisted of four sets of specific exercises (Figure 3). Each exercise was executed at maximal intensity for 20 s, followed by a 10-s recovery period, with a 2-min rest interval between sets. Participants in the BFR-Tabata group wore a 5 cm wide KAATSU pneumatic cuff (Kaatsu-Master, KAATSU Global, Japan) that was positioned around each thigh approximately 1–2 cm distal to the inguinal crease (Kim et al., 2012; Hori et al., 2022). The Tabata group followed the same training program but used textile straps that did not apply any effective pressure. Inflate approximately 20 s before the exercise begins, and deflate immediately after completing each set. Before training, we measured arterial occlusion pressure (AOP) at the posterior tibial artery with Doppler ultrasonography (Apogee 1,000). We chose this site because study show valid and reproducible results when using relative pressures with a proximal thigh cuff (Mouser et al., 2018). Participants were in a standing position during this assessment. A pneumatic cuff was then placed around the upper thigh and gradually inflated while the participant remained standing until the Doppler signal indicated a complete cessation of arterial blood flow. The compression pressure was set at 60% of each participant’s AOP, a mid-range value within the recommended 40%–80% range for lower-limb BFR (Patterson et al., 2019), which balances efficacy and tolerability and is consistent with prior evidence-based protocols (Gonzalez Rojas et al., 2024; Bourgeois et al., 2025). To monitor vascular adaptations, AOP was reassessed weekly to monitor vascular adaptations, and BFR pressure was set each week at 60% of that week’s AOP; detailed values are provided in Supplementary Table S1.

#### 2.2.2 Outcomes measurement

##### 2.2.2.1 Primary outcomes

###### 2.2.2.1.1 Wingate anaerobic test

Participants performed a 30-s Wingate anaerobic test on a stationary bicycle ergometer (Model 894E, Monark, Sweden) to obtain measures of peak power (PP), average power (AP), time to peak (TTP), and fatigue index (FI). Participants first completed a 10-min warm-up that included easy riding and dynamic stretching. After the warm-up, participants performed a 30-s all-out Wingate with the braking force set at 7.5% of body mass, consistent with prior studies (Yaprak, 2020; Ko et al., 2021). The assessors provided encouragement during the test to help participants perform their best. The test–retest reliability of the Wingate anaerobic test has been established in previous study, with ICC typically ranging from 0.85 to 0.98 across key performance variables such as PP, AP, and FI (Malone et al., 2014).

###### 2.2.2.1.2 Badminton-specific endurance test

**FIGURE 4:**
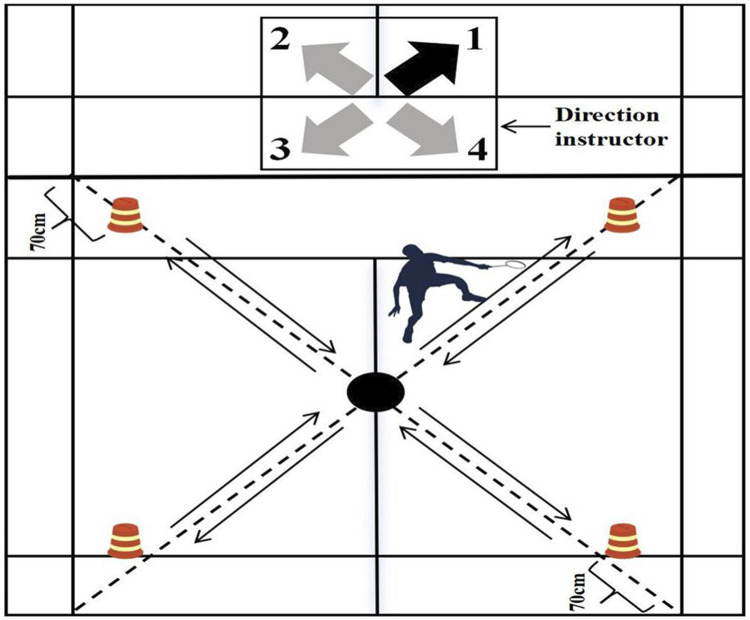
Badminton-specific endurance test.

We adopted a previously developed badminton-specific endurance test with established test–retest reliability: Ando et al. (2024) reported CVs of 11.9% for blood lactate and 1.5% for reach time, indicating good reproducibility (Ando et al., 2024). We set up four markers at designated locations on a standard badminton court. The participants began at the midpoint of the half-court and touched the markers in a specified order. The sequence was as follows: 1) move to the right front marker and return to the center point; 2) move to the left front marker and return to the center point; 3) move to the left back marker and return to the center point; 4) move to the right back marker and return to the center point. Participants repeated this sequence for 30 s, recording the total number of completed moves (Figure 4).

##### 2.2.2.2 Secondary outcomes

###### 2.2.2.2.1 Vertical jump test (SJ and CMJ)

We utilized a force platform (Kistler Group, Switzerland) to assess squat jump (SJ) and countermovement jump (CMJ) in participants. The test–retest reliability of both SJ and CMJ has been established in previous study. Markovic et al. (2004) reported high intraclass correlation coefficients (ICC = 0.97 for SJ and ICC = 0.98 for CMJ) (Markovic et al., 2004).

The SJ test was utilized to assess the concentric muscle contraction ability. Participants were instructed to maintain a static position with their knees flexed at a 90° angle, as verified by a dynamic joint goniometer. After a 2-s pause, they were required to perform a forceful vertical jump without any preparatory squatting movements (Van Hooren and Zolotarjova, 2017).

The CMJ test was used to assess the stretch-shortening cycle (SSC) ability. Participants began in an upright position and, upon hearing an audible signal, performed a knee flexion before jumping vertically to reach maximum height. They were instructed to land on a force platform positioned in the center. Additionally, participants were told to keep both hands fixed on their iliac crests throughout the exercise (gözlükaya Girginer et al., 2024).

Participants completed three maximal effort jumps, and the highest jump height was chosen for analysis in both the SJ and CMJ tests (Claudino et al., 2017). A standardized rest interval of 180 s was implemented between jumps. The assessors provided verbal feedback to help participants achieve their best performance.

### 2.3 Statistical analysis

All variables were tested for normality using the Shapiro–Wilk test, and the homogeneity of variances was assessed with Levene’s test. Data were presented as mean ± standard deviation (SD). Statistical analyses were performed using the SPSS statistical package (version 25.0, IBM Statistics, Chicago, IL). A two-way repeated measures ANOVA with a 2 (time: pre–post intervention) × 2 (group: experimental–control) design was conducted to examine both within- and between-group differences. The effect size was expressed as partial eta squared ( $\eta_{p}^{2}$ ), with thresholds defined as small (0.01 ≤ $\eta_{p}^{2}$ ≤ 0.06), moderate (0.06 ≤ $\eta_{p}^{2}$ < 0.14), and large ( $\eta_{p}^{2}$ ≥ 0.14) (Cohen, 2013). For significant interaction, pairwise comparison was conducted to find any possible mean differences, with their p-values adjusted via Bonferroni methods. In cases where the assumption of sphericity was violated, the Greenhouse-Geisser correction was applied. Cohen’s d (reported as absolute values) was calculated to quantify effect sizes (d) for within-group pre–post comparisons and between-group comparisons, with the following thresholds: trivial (d < 0.2), small (0.2 ≤ d < 0.6), moderate (0.6 ≤ d < 1.2), large (1.2 ≤ d < 2.0), very large (2.0 ≤ d < 4.0), and extremely large (d ≥ 4.0) (Hopkins et al., 2009). A p-value <0.05 was considered statistically significant.

## 3 Results

All 30 participants completed the training intervention, resulting in a 100% adherence rate. Throughout the intervention, no adverse events occurred, and no participants reported subjective discomfort. The results of Shapiro-Wilk and Levene’s test showed that when considering that the calculated significance value was more than five hundredths, the assumption of normality of data distribution and homogeneity of variances were met.

### 3.1 Primary outcomes

#### 3.1.1 Wingate anaerobic test

##### 3.1.1.1 PP

**FIGURE 5:**
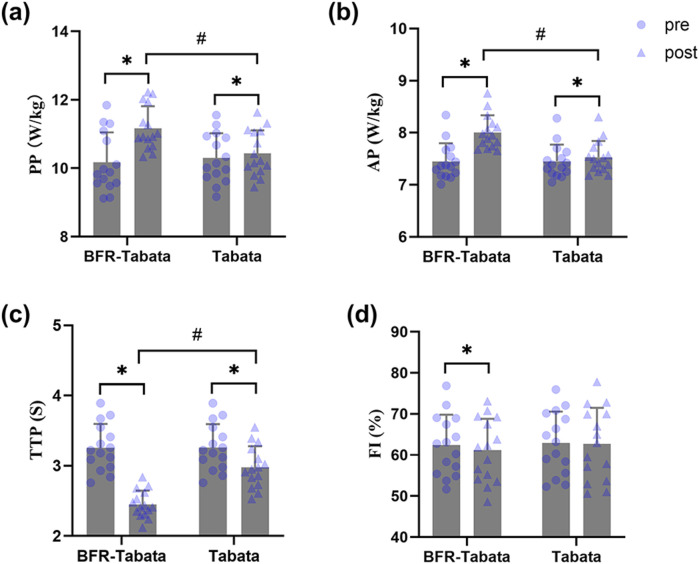
Effect of BFR-Tabata on the Wingate anaerobic test. Mean ± SD. **(a)** PP, peak power; **(b)** AP, average power; **(c)** TTP, time to peak; **(d)** FI, fatigue index. *: compared with pre significant difference; #: between-group significant difference.

There was a significant main effect of time (*F* = 165.91, *p* < 0.001, $\eta_{p}^{2}$ = 0.86) and interaction (F = 95.14, p < 0.001, $\eta_{p}^{2}$ = 0.77). After the training period, both groups significant improved (BFR-Tabata: p < 0.01, d = 2.99, 95% CI: [1.78 to 4.19]; Tabata: p = 0.04, d = 1.80, 95% CI: [0.96 to 2.62]), and a significant between-group difference was observed (p = 0.01, d = 1.11, 95% CI: [0.33 to 1.88]) (Figure 5a) (Table 1).

**TABLE 1:** Effects of BFR-Tabata training on anaerobic capacity in badminton players.

Variable	Group	Pre	Post	Cohen’s d (95% CIs)		RM ANOVA				
				Within-group	Between-group	Source	df	*F*	*p*	*η* ^*2*^
Vertical jump test	CMJ (cm)					Time × group	1, 28	58.82	<0.001	0.68
	BFR-Tabata	48.25 ± 5.07	51.05 ± 5.05	3.92 (2.93–5.43)	0.25 (-0.47–0.96)	Time	1, 28	193.98	<0.001	0.87
	Tabata	49.02 ± 5.04	49.83 ± 4.77	1.15 (0.48–1.80)		Group	1, 28	0.02	0.90	0.001
	SJ (cm)					Time × group	1, 28	74.04	<0.001	0.73
	BFR-Tabata	45.25 ± 6.71	47.21 ± 6.70	3.53 (2.13–4.91)	0.21 (-0.50–0.93)	Time	1, 28	74.04	<0.001	0.73
	Tabata	45.83 ± 6.63	45.83 ± 6.01	0.00 (−0.51 to 0.51)		Group	1, 28	0.03	0.87	0.001
Wingate anaerobic test	PP (W/kg)					Time × group	1, 28	95.14	<0.001	0.77
	BFR-Tabata	10.18 ± 0.87	11.17 ± 0.64	2.99 (1.78–4.19)	1.11 (0.33–1.88)	Time	1, 28	165.91	<0.001	0.86
	Tabata	10.31 ± 0.72	10.44 ± 0.67	1.80 (0.96–2.62)		Group	1, 28	1.31	0.26	0.05
	AP (W/kg)					Time × group	1, 28	369.06	<0.001	0.93
	BFR-Tabata	7.45 ± 0.35	8.01 ± 0.33	6.93 (4.34–9.52)	1.51 (0.68–2.32)	Time	1, 28	658.38	<0.001	0.96
	Tabata	7.45 ± 0.32	7.53 ± 0.31	1.47 (0.73–2.19)		Group	1, 28	4.05	0.05	0.13
	TTP (s)					Time × group	1, 28	186.33	<0.001	0.87
	BFR-Tabata	3.26 ± 0.33	2.45 ± 0.19	5.56 (3.46–7.65)	2.10 (1.19–2.99)	Time	1, 28	799.09	<0.001	0.97
	Tabata	3.26 ± 0.33	2.98 ± 0.30	8.18 (5.14–11.21)		Group	1, 28	6.25	0.02	0.18
	FI (%)					Time × group	1, 28	1.70	0.20	0.06
	BFR-Tabata	62.40 ± 7.40	61.13 ± 7.67	0.76 (0.17–1.33)	0.19 (-0.52–0.90)	Time	1, 28	3.72	0.06	0.12
	Tabata	62.95 ± 7.61	62.71 ± 8.78	0.10 (−0.41–0.60)		Group	1, 28	0.14	0.71	0.005
Badminton-specific endurance test	Badminton-specific endurance test (n)					Time × group	1, 28	18.18	<0.001	0.39
	BFR-Tabata	18.53 ± 1.19	21.13 ± 1.77	2.46 (1.42–3.49)	0.92 (0.16–1.67)	Time	1, 28	152.91	<0.001	0.85
	Tabata	18.33 ± 1.18	19.60 ± 1.55	2.13 (1.19–3.05)		Group	1, 28	2.97	0.096	0.09

M ± SD; P < 0.05, statistical significance; AP, average power; CMJ, countermovement jump; FI, fatigue index; PP, peak power; SJ, squat jump; TTP, Time to Peak.

##### 3.1.1.2 AP

There was a significant main effect of time (F = 658.38, p < 0.001, $\eta_{p}^{2}$ = 0.96) and interaction (F = 369.06, p < 0.001, $\eta_{p}^{2}$ = 0.93). After the training period, both groups significantly improved (BFR-Tabata: p < 0.001, d = 6.93, 95% CI: [4.34 to 9.52]; Tabata: p < 0.001, d = 1.47, 95% CI: [0.73 to 2.19]), and a significant between-group difference was observed (p < 0.001, d = 1.51, 95% CI: [0.68 to 2.32]) (Figure 5b).

##### 3.1.1.3 TTP

There was a significant main effect of time (F = 799.09, p < 0.001, $\eta_{p}^{2}$ = 0.97), group (F = 6.25, p = 0.02, $\eta_{p}^{2}$ = 0.18), and interaction (F = 186.33, p < 0.001, $\eta_{p}^{2}$ = 0.87). After the training period, both groups significantly improved (BFR-Tabata: p < 0.001, d = 5.56, 95% CI: [3.46 to 7.65]; Tabata: p < 0.001, d = 8.18, 95% CI: 5.14–11.21), and a significant between-group difference was observed (p < 0.001, d = 2.10, 95% CI: [1.19 to 2.99]) (Figure 5c).

##### 3.1.1.4 FI

The statistical analysis revealed no significant main effect of time for FI (F = 3.72, p = 0.06, $\eta_{p}^{2}$ = 0.12), no significant main effect of group (F = 0.14, p = 0.71, $\eta_{p}^{2}$ = 0.001), and no significant interaction effect (F = 1.70, p = 0.20, $\eta_{p}^{2}$ = 0.06) (Figure 5d).

#### 3.1.2 Badminton-specific endurance test

**FIGURE 6:**
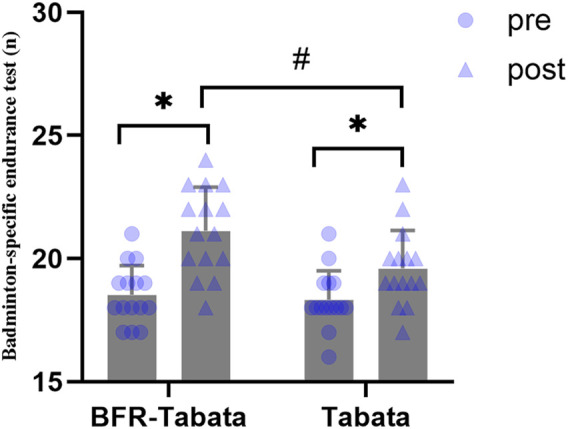
Effect of BFR-Tabata on the badminton-specific endurance test. Mean ± SD; *: compared with pre significant difference; #: Between-group significant difference.

There was a significant main effect of time (F = 152.91, p < 0.001, $\eta_{p}^{2}$ = 0.85) and interaction (F = 18.18, p < 0.001, $\eta_{p}^{2}$ = 0.39). After the training period, both groups significantly improved (BFR-Tabata: p < 0.001, d = 2.46, 95% CI: [1.42 to 3.49]; Tabata: p < 0.001, d = 2.13, 95% CI: [1.19 to 3.05]), and a significant between-group difference was observed (p = 0.02, d = 0.92, 95% CI: [0.16 to 1.67]) (Figure 6) (Table 1).

### 3.2 Secondary outcomes

#### 3.2.1 Vertical jump test

##### 3.2.1.1 SJ

**FIGURE 7:**
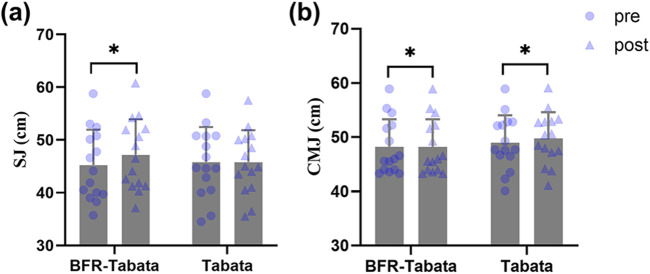
Effect of BFR-Tabata on the vertical jump test. Mean ± SD. **(a)** SJ, squat jump; **(b)** CMJ, countermovement jump. *: compared with pre significant difference; #: between-group significant difference.

There was a significant main effect of time (F = 74.04, p < 0.001, $\eta_{p}^{2}$ = 0.73) and interaction (F = 74.04, p < 0.001, $\eta_{p}^{2}$ = 0.73). After the training period, the BFR-Tabata group significantly improved (p < 0.001, d = 3.53, 95% CI: [2.13 to 4.91]), whereas the Tabata group showed no significant change (p = 0.99, d = 0.00, 95% CI: [-0.51 to 0.51]), and no significant between-group difference was observed (p = 0.56, d = 0.21, 95% CI: [-0.50 to 0.93]) (Figure 7a) (Table 1).

##### 3.2.1.2 CMJ

There was a significant main effect of time (F = 193.98, p < 0.001, $\eta_{p}^{2}$ = 0.87) and interaction (F = 58.82, p < 0.001, $\eta_{p}^{2}$ = 0.68). After the training period, both groups significantly improved (BFR-Tabata: p < 0.001, d = 3.92, 95% CI: [2.39 to 5.43]; Tabata: p < 0.001, d = 1.15, 95% CI: [0.48 to 1.80]), and no significant between-group difference was observed (p = 0.50, d = 0.25, 95% CI: [-0.47 to 0.96]) (Figure 7b).

## 4 Discussion

To the best of our knowledge, this is the first study to investigate the effects of 6 weeks of BFR-Tabata training on anaerobic capacity in male badminton players. The findings indicate that BFR-Tabata training significantly enhances specialized anaerobic endurance compared to Tabata training alone, but has limited enhancement of lower-limb explosive strength.

The improvement in badminton-specific endurance performance may be attributable to enhancements in anaerobic endurance. Previous studies indicate that BFR training can induce localized hypoxia, increasing reliance on anaerobic glycolysis and elevating blood lactate concentration. (Amani-Shalamzari et al., 2020; Thompson et al., 2024; Li et al., 2023; Y et al., 2021). For instance, Thompson et al. (2024) demonstrated that 4 weeks of BFR-rowing intervention significantly increased lactate levels in elite rowers (Thompson et al., 2024); while Amani-Shalamzari et al. (2020) similarly reported higher blood lactate concentrations in BFR groups compared to controls (Amani-Shalamzari et al., 2020). Meanwhile, chronic BFRIT has been reported to delay the onset of blood lactate accumulation (OBLA) (de Oliveira et al., 2016), suggesting improved lactate clearance and, consequently, greater lactate tolerance. This process may involve adaptations in glucose uptake and mitochondrial function. Relevant fundamental research has proposed that the hypoxia-induced upregulation of glucose transporter type 4 (GLUT4) caused by BFR plays a pivotal role in enhancing muscle glucose extraction, thereby promoting glycogen storage, facilitating cross-membrane glucose transport, improving lactate metabolism (Burgomaster et al., 2003; Christi et al., 2019). Therefore, we speculate that the observed increase in anaerobic capacity in this study may be related to enhanced lactate tolerance. Current research primarily focus on peripheral mechanisms (Chua et al., 2022; Yin et al., 2025), with the FI utilized in this study predominantly reflecting peripheral fatigue. Future research should explore central mechanisms, including neural activation and changes related to electroencephalography, to enhance our understanding of the anti-fatigue effects of BFR training.

The finding of this study is consistent with previous studies that found BFRIT to be effective in enhancing AP in athletes (Amani-Shalamzari et al., 2020; Bourgeois et al., 2025; Park et al., 2010). Amani-Shalamzari et al. (2020) used BFR in small-sided games (SSG) training, resulting in improved AP in Futsal players after 3 weeks (10 sessions) (Lavigne et al., 2024). This is supported by a recent report from Bourgeois et al. (2025), which found that 3 weeks of BFRIT significantly improved both AP and total work in male endurance athletes (Bourgeois et al., 2025). However, McKee et al. (2023) suggested that less than 6 weeks of BFRIT do not improve AP in amateur male team-sport players (Mckee et al., 2023). A meta-analysis showed that training protocols (e.g., training durations, training status) are moderators that influence physiological adaptations in BFRIT-enhanced athletes (Yin et al., 2025). Future research should investigate how training status (e.g., elite and amateur athletes) and durations (e.g., greater than 6 weeks or less than 6 weeks) affect training outcomes.

This study found that BFRIT was able to improve PP, which is in contrast to previous studies. A meta-analysis indicated that BFRIT did not show a statistically significant benefit over traditional interval training (IT) in enhancing PP(50). Taylor et al. (2016) and Mitchell et al. (2019) showed similar PP improvements in both BFR and control groups, with no significant between-group differences. The training duration (e.g., 3–4 weeks) and number of sessions used in previous studies (Mitchell et al., 2019; Taylor et al., 2016) may not be adequate to increase PP compared to our training protocol. This study used the BFR-Tabata protocol, featuring eight 20-s all-out efforts with 10-s rests. This approach led to a higher training intensity than previous SIT protocols (Mitchell et al., 2019; Taylor et al., 2016), which consisted of 30-s sprints followed by 4.5-min recoveries. The BFR pressure setting may affect training effectiveness. This study employed individualized pressure settings based on each participant’s arterial occlusion pressure (AOP), thereby standardizing the relative restriction stimulus across individuals. In contrast, previous studies implemented fixed pressure protocols (Mitchell et al., 2019; Taylor et al., 2016), which may have introduced inter-individual variability in vascular occlusion, potentially attenuating the efficacy and consistency of the BFR stimulus (Patterson et al., 2019).

This study observes that the BFR-Tabata group demonstrated a greater improvement in TTP than the Tabata group. We speculate that this enhancement may be related to greater recruitment of type II fibers and muscle hypertrophy under BFR, as previous research has shown that BFR training can enhance neural activation and facilitate type II motor unit engagement (Wang et al., 2023). Furthermore, the temporary increase in growth hormone (GH), insulin-like growth factor 1 (IGF-1), and testosterone from BFR creates an anabolic environment for muscle growth (Y et al., 2021; Li et al., 2022). Interestingly, our study found no significant changes in the FI for either group. One plausible explanation is that the Wingate test, as a single maximal effort, may not effectively reflect adaptations in fatigue resistance typically induced by interval-based training (W et al., 2025). These modalities primarily enhance recovery between repeated bouts rather than sustaining power in a single all-out effort (W et al., 2025). Thus, the lack of change in FI likely reflects a mismatch between training adaptations and the test characteristics.

Another important finding was that BFR-Tabata did not further enhance lower limb explosive strength compared to previous studies (Amani-Shalamzari et al., 2020; Park et al., 2010; Mckee et al., 2023; Elgammal et al., 2020; Tangchaisuriya et al., 2021). This discrepancy could be attributed to interval recovery time. Using a 10-s recovery period during BFR-Tabata training may be detrimental to skeletal muscle anabolism. Amani-Shalamzari et al. discovered that combining BFR with SSG training, which consists of 3-min repetitive sprints followed by 2-min rests, effectively improved athletes’ muscular strength (Amani-Shalamzari et al., 2020). Elgammal et al. observed comparable findings when implementing a 4-week BFR + repeated sprint training (RST) program consisting of three weekly sessions (Elgammal et al., 2020). SSG and RST typically involve interval recovery time, which increase muscle strength adaptations (Yin et al., 2025). Sufficient recovery time between intervals helps reduce skeletal muscle fatigue and enhances explosiveness in the lower extremities (Loenneke et al., 2015). In contrast, the BFR-Tabata protocol may have resulted in insufficient cumulative ischemic stimulus, thereby attenuating the extent of muscle adaptation. This is supported by the findings of Abe et al. (2009), who demonstrated that performing BFR walking once daily yielded approximately half the improvements in muscular strength and hypertrophy compared to a twice-daily training frequency (Abe et al., 2009). De Oliveira et al. reported that although the BFRIT group incorporated BFR during training, the BFR component accounted for only 50% of the training volume compared to the BFR-only group. This reduced exposure likely resulted in insufficient cumulative mechanical and metabolic stress, thereby failing to elicit significant improvements in muscle strength (de Oliveira et al., 2016). In summary, the choice of training modality should be closely matched to the targeted adaptation. For a training goal aimed at enhancing lower limb explosive strength, the BFR-Tabata mode may not be sufficient. Future research could further explore the effects of combining different intermittent durations with BFR. It has been shown that BFR combined with resistance training exhibits more significant effects in enhancing dynamic muscle strength (Moore et al., 2004; Yasuda et al., 2011).

## 5 Limitations

The interpretation of the findings should consider potential limitations. First, while various mechanisms, such as metabolic stress, fast-twitch fiber recruitment, and hormonal regulation, were suggested, no physiological markers were measured directly. For better validation, future studies should include muscle biopsies, enzyme activity assessments, and hormone level evaluations. The absence of acute-session blood lactate and standardized intensity metrics (e.g., %V̇O_2max_, %HRmax, RPE) limits the physiological interpretation of our findings. Additionally, this study included only male participants, limiting generalizability; given the larger hormonal fluctuations in females that can influence experimental outcomes, future research should include female participants. Furthermore, the 6-week intervention period may not have been sufficient to achieve significant physiological adaptations. It is advisable to extend the training duration to obtain more comprehensive training effects. This study is underpowered for detecting interaction effects and smaller outcomes, so it should be interpreted cautiously and confirmed in larger samples. Despite these limitations, this study provides practical insights into BFR-Tabata as an effective training method for badminton players.

## 6 Conclusion

In conclusion, BFR-Tabata training represents a promising and practical strategy for optimizing anaerobic endurance performance in badminton players. Its capacity to elicit targeted physiological adaptations makes it particularly suitable for elite athletes engaged in high-intensity, intermittent sports, supporting its integration into advanced training programs. However, it has a limited effect on lower limb explosive strength (CMJ/SJ) enhancement. Further research is needed to explore the underlying physiological and molecular mechanisms of BFR-Tabata training, including hormonal responses, muscle oxygenation, and metabolic biomarkers, to better understand its specific adaptive pathways in elite athletes.

## Data Availability

The original contributions presented in the study are included in the article/Supplementary Material, further inquiries can be directed to the corresponding authors.
